# Proposal and Implementation of a Procedure for Compliance Recognition of Objects with Smart Tactile Sensors

**DOI:** 10.3390/s23084120

**Published:** 2023-04-19

**Authors:** Raúl Lora-Rivera, Óscar Oballe-Peinado, Fernando Vidal-Verdú

**Affiliations:** 1 Instituto de Investigación Biomédica de Málaga (IBIMA), Universidad de Málaga (UMA), 29010 Malaga, Spain; 2 Instituto Universitario de Investigación en Ingeniería Mecatrónica y Sistemas Ciberfísicos (IMECH.UMA), Universidad de Málaga (UMA), 29017 Malaga, Spain; oballe@uma.es (Ó.O.-P.); fvidal@uma.es (F.V.-V.)

**Keywords:** tactile sensors, object recognition, FPGA implementation, real-time feature extraction

## Abstract

This paper presents a procedure for classifying objects based on their compliance with information gathered using tactile sensors. Specifically, smart tactile sensors provide the raw moments of the tactile image when the object is squeezed and desqueezed. A set of simple parameters from moment-versus-time graphs are proposed as features, to build the input vector of a classifier. The extraction of these features was implemented in the field programmable gate array (FPGA) of a system on chip (SoC), while the classifier was implemented in its ARM core. Many different options were realized and analyzed, depending on their complexity and performance in terms of resource usage and accuracy of classification. A classification accuracy of over 94% was achieved for a set of 42 different classes. The proposed approach is intended for developing architectures with preprocessing on the embedded FPGA of smart tactile sensors, to obtain high performance in real-time complex robotic systems.

## 1. Introduction

Tactile sensors are increasingly being used in a variety of applications. This has led to the development of electronic skin (e-skin) devices, which open up possibilities for healthcare, human–machine interfaces, virtual reality, artificial intelligence-related, and robotic applications [1]. In this context, object manipulation and interaction with the environment involves the detection of properties such as the friction coefficient, texture, geometry, and stiffness [2,3].

Stiffness can be estimated in a fairly straightforward manner from measurements of the force and displacement when the object is squeezed [4]. However, the force–displacement curve of a compliance object can be far from linear, being quite complex instead [5]. In reference [5], FPCA was used to approximate the force–displacement curve obtained from two pressure sensors with three basis functions. Eight different soft objects were classified, and the best results were obtained with a *k-NN* classifier. Eight parameters were computed from the readings of two force sensors when a vegetable was squeezed until it collapsed in [6]. These parameters were the mean value, variance and standard deviation, maximum force, quality factor, quartiles, and quartile factor. All data recorded from the two sensors were reduced to these eight parameters, and a decision tree algorithm obtained good results in classifying tomatoes into three categories depending on their stiffness. Hardness can also be estimated from analysis of data such as the reaction force when an object is explored using some predefined motions [7,8].

On the other hand, the compliance of an object can be estimated from the output of tactile sensors, taking advantage of advanced artificial hands and grippers equipped with them. The whole tactile image can be the input of complex neural network architectures such us convolutional neural networks (CNN), which estimate the stiffness from the image sequences taken while the object is deformed [9,10]. In these cases, the preprocessing of the tactile images is essentially filtering, normalization, and reshaping, to adapt them to the neural network input.

Another approach consists in using certain features from the tactile images instead of the whole image. This reduces the volume of data to be recorded and processed, and simplifies the computation. The maximum force measured by a taxel (force sensing unit in the tactile sensor), the area of contact, and the displacement of its centroid were used in [11]. Different combinations of features and algorithms were explored and good results were obtained with hidden Markov models, to classify eight objects into four categories.

The first two colour moments of the tactile image, i.e., the mean value and the standard deviation of the taxels, were proposed as features in [12]. A *k-NN* classifier was used with the distance computed with the dynamic time warping algorithm (DTW). A similar strategy was followed in [13], with a tactile sensor focused on a large compliance and the displacement of their taxels.

Previous proposals achieved a good performance with a limited number of objects and categories. However, though the proposed features reduced the data storage and the computation complexity, both remain high in the case of embedded systems. These systems can perform distributed computation with smart sensors, which improves the latency and reduces data traffic in communication buses. A smart tactile sensor based on an embedded FPGA and direct sensor-FPGA interface was proposed in [14]. This sensor provides the first raw moments of the tactile image. These moments give information about the area of contact, contact force, location, shape, and orientation of the tactile image. Therefore, the time series of these moments taken while an object is being squeezed and de-squeezed provides much information about how it is deformed. Use of the entire registered series of moment values is proposed by the authors of this paper, as a feature vector for the classifier detailed in the conference paper in [15], where nine objects were classified with up to 86.7% accuracy. Since the thus-obtained feature vector is large, here we propose a further step, aimed at reducing the complexity and data management required, which consists in extracting features similar to those used in realizations not based on tactile sensors [16,17]. In this way, complex computations such as the DTW distance of the whole moment series are not required, only the Euclidean distance of the feature vector whose components are the selected features, which is much simpler. This approach was implemented on a system on chip-based architecture and many different options were analyzed, in terms of the performance and complexity during the task of the classification of a set of 42 classes.

The rest of this paper is organized as follows: Section 2 proposes the feature extraction algorithm. Section 3 describes the materials and methods. Section 4 is devoted to the implementation of the approach on a system on chip. Finally, the results and related discussions are detailed in Section 5, while Section 6 summarizes the main conclusions of the work and considers future extensions.

## 2. Proposed Features for Classification

As mentioned in the Introduction, the raw moments of the tactile image are computed locally in the robotic finger and the palm [18,19]. In particular, the {p,q} order of moments of the tactile image is computed as follows:

(1) $$M_{p,q}=\sum_{x=1}^{N}\sum_{y=1}^{M}x^{p}y^{q}I\left(x,y\right)$$

where I(x,y) is the output from the *taxel* at row *x* and column *y*.

**Figure 1:**
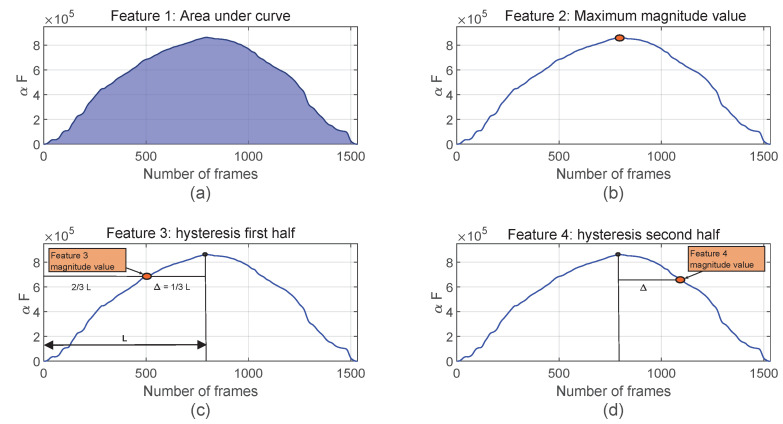
Proposed features for the moment $M_{0,0}$ and the object #OBJ-2 (potato): (**a**) area under the curve (AR feature), (**b**) maximum value (MAX feature), (**c**) magnitude value in the ascending part at 2/3 of the time to reach the maximum (H1 feature), and (**d**) magnitude value at 1/3 of the same time after the maximum is reached (H2 feature).

The raw moments of the tactile images versus time curves that are registered as the objects are compressed or decompressed. Figure 1 shows these curves for an example object (potato) and moment ($M_{0,0}$). The following set of features are defined from this graph, to significantly reduce the consumption of resources and the dimensions of the classifier input feature vector:

- AR (Figure 1a): Area under the raw moment curve;
- MAX (Figure 1b): Magnitude value at the maximum displacement;
- H1 (Figure 1c): Magnitude value before one third of the time needed to reach the peak of the curve;
- H2 (Figure 1d): Magnitude value after one third of the time to needed reach the peak of the curve.

H1 and H2 are related to the object hysteresis, which can be significant in objects made of elastomers. This hysteresis causes the graph to not be symmetric with respect to the axis defined by the peak. The three times related to the features MAX, H1, and H2 are known in advance, since the whole sequence is controlled by the robotic system. Therefore, the corresponding features do not require any computation and can be directly registered. Moreover, the area under the curve only requires a cumulative sum.

Note that there are six different raw moment curves ($M_{p,q}$) for each exploration, which involves both the finger (f) and the palm (p). When we consider the raw moments for both sensors and the four feature values (AR,MAX,H1,H2), there are a total of 48 features for each exploration. To avoid the issue of having redundant information, we included the option of reducing the data dimensions using principal component analysis (PCA) techniques.

## 3. Materials and Methods

In this section, we start by introducing the artificial finger and palm sensors used to gather the tactile data. Then, we describe the experimental setup and the objects of study. Afterwards, we explain the procedure we followed to collect the moment sequences during the explorations and the training algorithm used to classify the objects.

### 3.1. Sensors Technology

**Figure 2:**
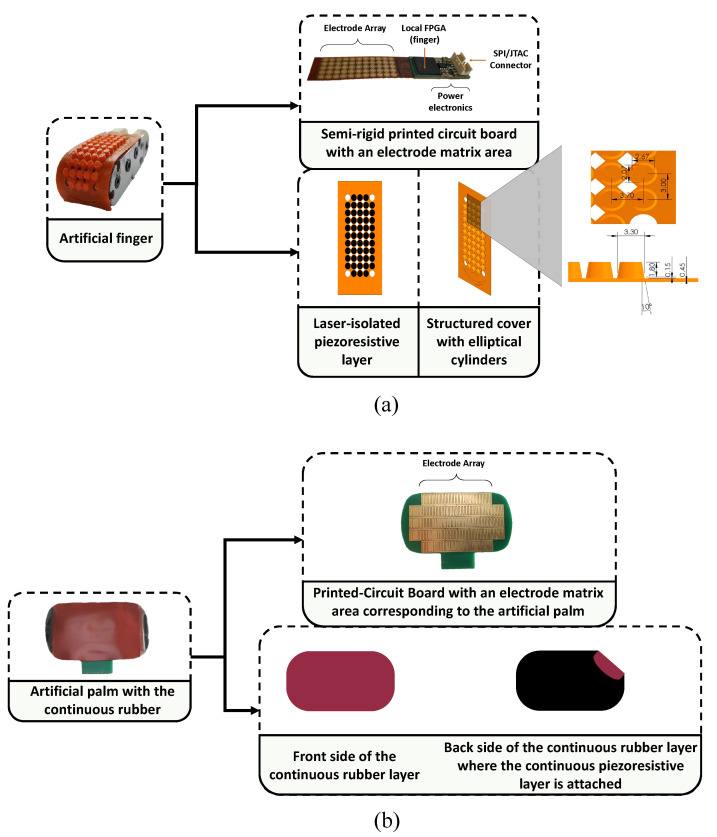
(**a**) The artificial finger is made of a semi-rigid printed circuit board, with a structured cover that helps to concentrate the force on the taxels (force sensing units in the tactile array). (**b**) Artificial palm with continuous cover. Dimensions are given in mm.

In this study, we acquired tactile data using two smart sensors from the tactile suite of the artificial hand reported by the authors in [19] (see Figure 2). The artificial finger shown in Figure 2a is equipped with tactile sensors made of a laser-isolated piezoresistive layer on an array of electrodes. The outer layer or cover is made of a thermoplastic elastomer (Filaflex^®^), and it has one dome per taxel in the tactile array. This design helps to concentrate the force and reduce the crosstalk between taxels. The sensor is capable of registering changes in the area and size of the tactile image when the objects are pressed and deformed. The spatial resolution of the sensor, that is, the minimum distance between two taxels, is 3.7 mm, and the size of the sensor is 40.7×15.0 mm. The electronics of the sensor are based on an FPGA (Spartan-6^®^). Data are sampled at a frequency of $F_{s}=485$ Hz, or a sampling period of $T_{s}=2.06$ ms.

The artificial palm in Figure 2b was built in a similar way. However, unlike for the finger, the palm sensor does not require laser isolation of the piezoresistive material, because the crosstalk is reduced by the electronics. Therefore, a cover made of a continuous rubber attached to a continuous piezoresisitve layer is placed atop the electrodes. The palm sensor electronics were also based on a Spartan-6^®^ FPGA. It implements the interface with the palm raw tactile sensor and also communicates with the finger sensor through an SPI serial bus, and with a personal computer through USB.

### 3.2. Experimental Setup

**Figure 3:**
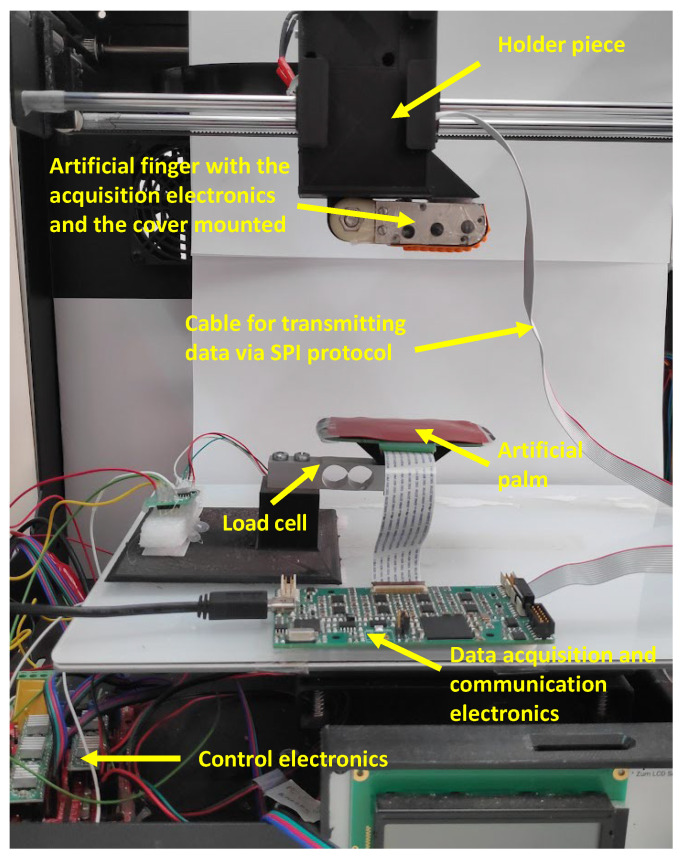
Experimental setup with the artificial finger and palm.

Figure 3 shows the experimental setup built to perform the object explorations. The artificial finger with the smart tactile sensor is at the top of Figure 3, while the palm is placed at the bottom. A strain gauge is used to provide a reference value of the force exerted by the palm. The finger is moved in the vertical axis using a motor controlled via an Arduino Mega 2560^®^ board, so that a compression–decompression movement between the finger and palm can be carried out. As said above, the tactile data and locally computed moments are gathered by the finger and palm electronics and sent to the computer via USB.

### 3.3. Objects to Explore

**Figure 4:**
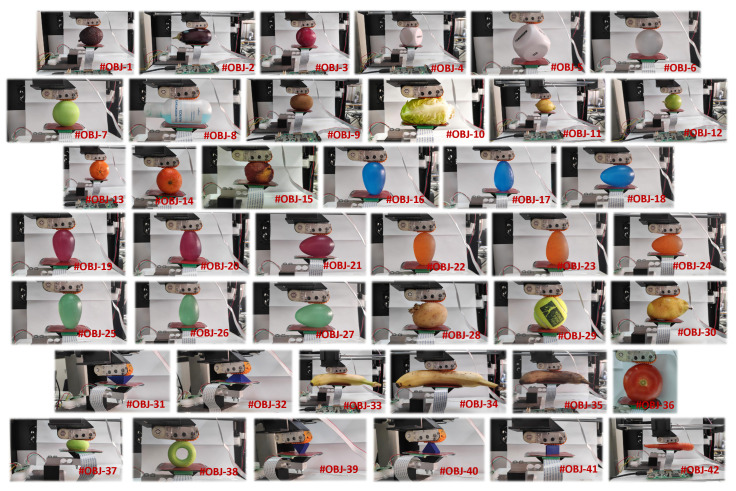
Set of 42 object classes used to carry out the palpation exploration, also see Table 1.

We selected 42 objects with different shapes and compliances (see Figure 4 and Table 1), which were grouped into four sets. The first set consisted of 3D printed objects (#OBJ-7, #OBJ-31, #OBJ-32, and #OBJ-37 to #OBJ-41) made of the flexible thermoplastic materials Filaflex^®^ and TPU^®^. The second set comprised hand-therapy grip 3D ovoids of varying stiffness (#OBJ-16 to #OBJ-18 with middle-high stiffness; #OBJ-19 to #OBJ-21 with high stiffness; #OBJ-22 to #OBJ-24 with low stiffness; #OBJ-25 to #OBJ-27 with middle-low stiffness). The third set consisted of objects selected from real-world scenarios (#OBJ-4, #OBJ-5, #OBJ-6, and #OBJ-29), some of which were used in different positions. Last, to consider applications such as smart agriculture and food processing, we included 17 pieces of fruits and vegetables in varying states of preservation: immature, mature, or rotten (#OBJ-1, #OBJ-2, #OBJ-3, #OBJ-9 to #OBJ-15, #OBJ-28, #OBJ-30, #OBJ-33 to #OBJ-36, and #OBJ-42).

### 3.4. Data Gathering Procedure

The following procedure was performed to obtain data for training and testing:

- Step 1: An object from the set shown in Figure 4 and Table 1 was manually placed between the artificial finger and the palm;
- Step 2: The palm was moved vertically to grasp the object, until the load cell detected a low-level threshold force of $F_{init}=0.1\;N$. This position was recorded as the initial point;
- Step 3: The palm was moved further vertically, so that the object was compressed until the palm reached a maximum relative distance from the initial point of approximately ≈1.2 cm (although the palm–finger gripper had a certain compliance, this limit was forced to avoid damage to the system when rigid objects were explored);
- Step 4: The palm was moved vertically in the reverse direction, so that the object was decompressed until the initial position defined in the Step 2 was reached.

**Figure 5:**
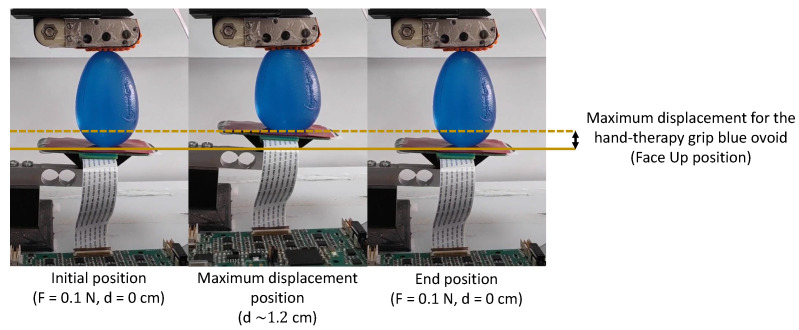
Illustration of the exploration sequence for the object class #OBJ-17.

This procedure was repeated at a velocity of v=10 mm/s and 47 times per object. During the squeeze–desqueeze sequences, the finger and palm tactile sensors collected data in real time, and they were sent to a personal computer and saved in text files using the Labview^®^ application. Figure 5 provides an overview of the methodology for the exploration of an object #OBJ-17.

**Figure 6:**
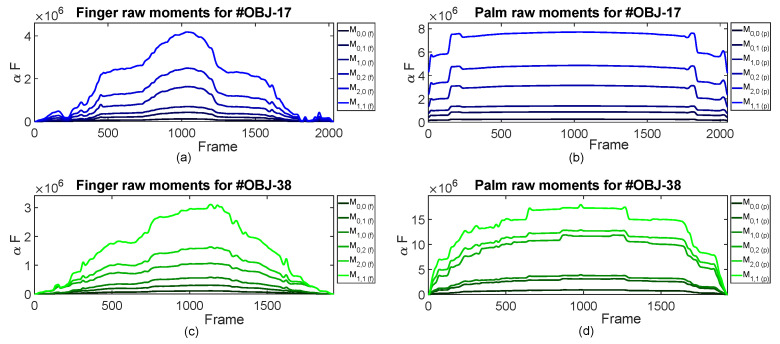
Curves obtained for the first six raw moments ($M_{p,q}$) obtained with the finger (**a**,**c**), and palm (**b**,**d**) from the exploration of the #OBJ-17 and #OBJ-38 classes.

Once the experiments had been performed with the 42 objects shown in Figure 4, we obtained the first six raw moments curves for the tactile images (1) of these objects. Figure 6 displays the example six first raw moment $M_{p,q}$ curves for the finger and palm and for the #OBJ-17 and #OBJ-38 objects in Figure 4 and Table 1, respectively.

### 3.5. Training Algorithm

This study utilized an unsupervised k-means classifier to obtain the results. The k-means classifier was selected due to its simplicity and speed of convergence compared to other machine-learning techniques such as support vector machines (SVM) or K-nearest neighbors (KNN), which require a significant amount of data storage for real-time tasks. The k-means++ method [20] was used to initialize the centroids, which represented the different classes, either randomly or in a systematic manner. The training process involved updating the centroids in response to the presented training set. This process was repeated with shuffled data until the centroids did not change or a maximum number of iterations was reached. The final trained classifier was selected based on the best accuracy achieved over one hundred repetitions of the whole procedure, using data from the test set [21].

## 4. Implementation on the Zynq7000^®^ SoC

**Figure 7:**
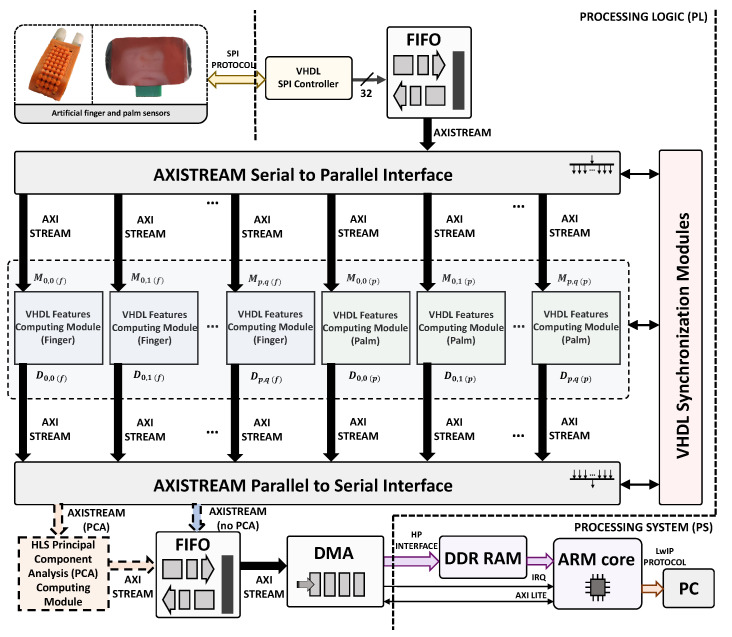
General architecture of the implementation on the Zynq-7000 XC7Z020^®^. The dashed part in orange is implemented if PCA is applied.

To demonstrate the feasibility of the approach and estimate its performance, a two step procedure was followed. First, data gathering was carried out using the finger and palm sensors, as described in Section 3.4, and the obtained moment–time graphs were transferred to the external DDR3 memory of an AVNET^®^ ZedBoard™ development board. This board is based on the Zynq™-7000 System on Chip (SoC) XC7Z020-CLG484-1 device, which has an FPGA and an ARM^®^ dual-core Cortex™ A9 processor. The feature extraction procedure described in Section 2 was then implemented on the FPGA of this SoC, while the classification algorithm was implemented in the ARM core. The use of this development board added flexibility for assessing the different alternatives before the implementation of a final system, where the features from the finger and palm sensors were transferred directly to a specific board with an embedded processor that implemented the classifier. Figure 7 illustrates this system and the overall datapath and processing logic actually implemented on the FPGA of the SoC. The FIFO (first-input first-output) output (32-bit words) was transmitted to an AXISTREAM Serial to Parallel Interface, which utilized the AMBA^®^ AXI-Streaming protocol for the communication between the FPGA hardware modules in the system. This allowed the input data (raw moments $M_{p,q}$ of the finger and palm) from the FIFO to be distributed to multiple preprocessing modules (VHDL Features Computing Module in Figure 7), while synchronization signals ensured their parallel execution in hardware. This is beneficial as we are maximizing the capabilities of the FPGA. In a scenario where both the finger and palm, and the maximum number of raw moments and features were utilized, the execution time remained unchanged compared to using a single module. This could be particularly useful for situations where the complexity of the system is increased.

The Vivado Design Suite™ environment was used to implement the preprocessing modules in Figure 7). This software integrates a hardware-description code written in VHDL/Verilog, as well as presynthetized cores from IP libraries or from the High-Level Synthesis (HLS) tool. The VHDL Features Computing Module blocks produce synchronized output data ($D_{p,q}$) for the finger and palm, respectively. These data are then transmitted to an AXISTREAM Parallel to Serial Interface through the use of the AMBA^®^ AXI-Streaming protocol. The feature vectors are serially transferred to a DDR memory for storage via a FIFO buffer and a DMA module. This setup allows the ARM core to access the stored data without having to manage data traffic. The FIFO output module allows frequency decoupling between the processing logic (PL) and processing system (PS) parts.

**Figure 8:**
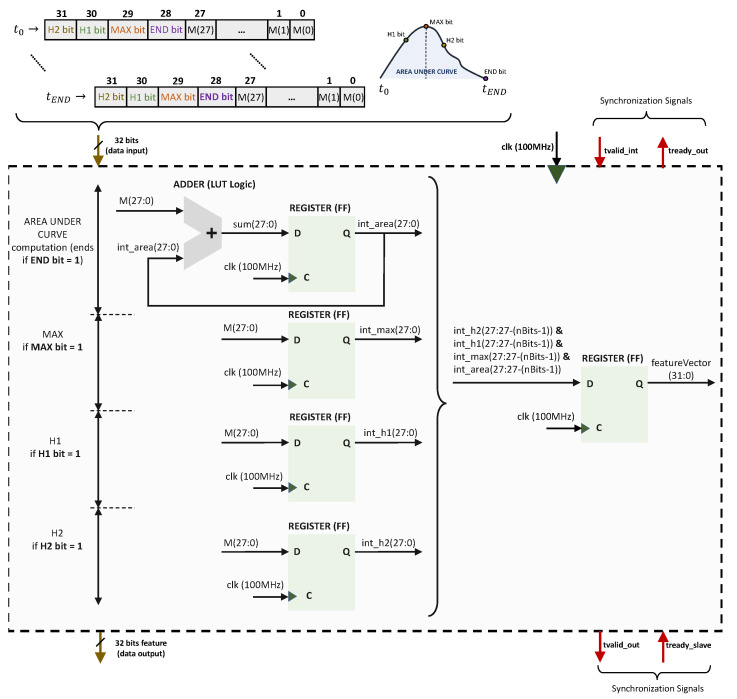
Proposed implementation of the VHDL Features Computing Module in Figure 7.

Figure 8 illustrates the obtention of the AR, MAX, H1, and H2 features from the p,q order moment of the tactile images (Equation (1)) the VHDL Features Computing Module. The instants required to read the values of H1, MAX, and H2 are provided in a field of the 32-bit input data (they are determined by the gripper controller in the squeeze–desqueeze sequence), and their reading only requires simple comparisons implemented in LUT Logic. On the other hand, the AR feature is computed with an adder that takes the 32-bit input data per clock cycle and adds this to the aggregated summation stored in a register. The values of the features are then concatenated into a single 32-bit output. This module also uses the *t_valid* and *t_ready* signals from the AMBA^®^ AXI-Streaming protocol for input/output synchronization. The clock frequency is $f_{clk}=100$ MHz. The feature output is provided only three clock cycles after the last input vector is read.

**Figure 9:**
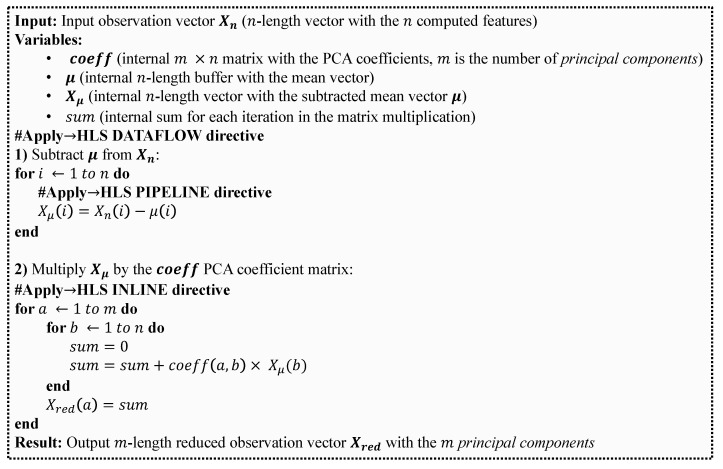
Pseudocode for the HLS principal component analysis (PCA) computing module in Figure 7.

An optional module to perform PCA is included in Figure 7. The inclusion of this module requires more memory and logical resources (Section 5), but a smaller number of features can be sent to the classifier implemented in the ARM core. The HLS principal component analysis (PCA) computing module implementation in Figure 7 was developed using System C in Vivado^®^ HLS. The corresponding pseudocode is shown in Figure 9. The Vivado optimization directive #HLS DATAFLOW enhances the concurrency of the RTL implementation. The pipeline implementation at RTL level is achieved through the use of #HLS PIPELINE and #HLS INLINE Vivado optimization directives. For each new $D_{p,q}$ feature vector, the pre-computed μ vector is subtracted and then, this result is multiplied by the PCA matrix coefficients **coeff**.

Finally, the k-means classifier (Section 3.5) was implemented in the ARM core using the SDK^®^ (System Development Kit) from Xilinx^®^. For this work, the classifier was trained offline and the calculated centroids were stored in the memoy of the SoC. High-speed communication with a personal computer was achieved using a real-time operating system (RTOS) and a lightweight TCP/IP stack, both implemented in the ARM core.

## 5. Results and Discussion

This section shows the results obtained from the object palpations described in Section 3. The processes of data gathering, feature extraction, and training for classification were explained in Section 3.4, Section 2, and Section 3.5, respectively.

### 5.1. Results Obtained without PCA

In order to determine the best implementation, we needed to consider different combinations of sensors (finger, palm), features (see Table 2), numbers of image moments per sensor, and bits per feature. Matlab^®^ was used to obtain the results in Table 3, which shows the highest accuracy percentage achieved without PCA, for different combinations of $M_{p,q}$ moments, features, and bits per feature (nbits/feature), respectively.

To assess the consumption of resources, we first identified cases with high accuracy (bolded in Table 3). These selected cases were implemented on the Zynq™-7000, and the consumption of resources was taken from the Utilization Report provided by the Vivado IDE^®^. The results are shown in Table 4.

As shown in Table 4, only a limited number of memory elements (distributed memory (LUTRAM), embedded block RAMs (BRAM), and flip-flop pairs (FF)) were required for these implementations. Specifically, the implementation only required slice registers (Flip-Flops). It is also worth noting that neither BRAMs nor Embedded DSPs (DSPs) were used in the computation of the features, which would make the migration of this design to other FPGA platforms easier.

At this point, to compare different options, we proposed the use of the figure of merit defined in (2).

(2) $$f\left(\alpha ,d,nBits,r\right)=\frac{\alpha }{d\cdot nBits\cdot r}$$

where α corresponds to the classification accuracy, *d* is the total number of input features for the classifier, nBits is the number of bits per feature, and *r* is the total number of hardware resources, as defined in (3).

(3) $$\begin{array}{cc} r=\frac{1}{6}\cdot & \left[\frac{\mathrm{LUTRAM}}{\max(\mathrm{LUTRAM})}+\frac{\mathrm{BRAMs}}{\max(\mathrm{BRAMs})}+\frac{\mathrm{FF}\mathrm{Pairs}}{\max(\mathrm{FF}\mathrm{Pairs})}+\frac{\mathrm{DSPs}}{\max(\mathrm{DSPs})}+\right.\\ & \left.+\frac{\mathrm{LUT}\mathrm{Logic}}{\max(\mathrm{LUT}\mathrm{Logic})}+\frac{F\text{-}\mathrm{MUXES}}{\max(F\text{-}\mathrm{MUXES})}\right] \end{array}$$

where max(LUTRAM), max(BRAMs), max(FF Pairs), max(DSPs), max(LUT Logic), and max(F-MUXES) are the maximum number of LUTRAM, BRAMs, FF Pairs, DSPs, LUT Logic, and F-MUXES in the Zynq™-7000 System on Chip (SoC) XC7Z020-CLG484-1 device, respectively.

**Figure 10:**
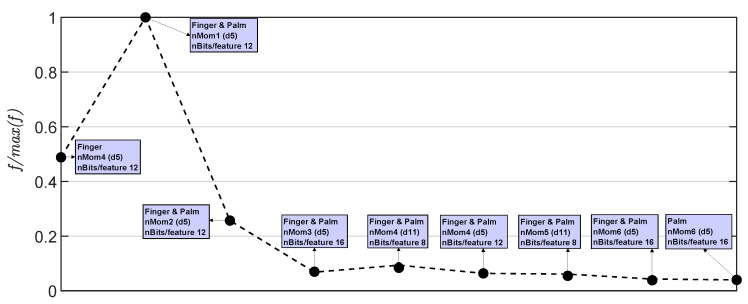
Result of the figure of merit in (2) normalized with respect to the best case.

Figure 10 displays the figure of merit for each case in Table 4. The labels in the figure show the corresponding case and its classification accuracy. The best performance in terms of hardware resources and accuracy percentage was achieved by the case Finger&Palm (nMom1-12bits/feature-d5). This is because it required fewer resources compared to the other implementations, which used more moments per sensor (right side of Figure 10). These other implementations achieved a high accuracy but consumed more hardware resources. In contrast, the Finger&Palm (nMom1-12bits/feature-d5) implementation only used the first raw moment for both the finger and palm, the d5 feature (combination of AR and MAX features in Table 2), and 12 bits per feature, while achieving a classification accuracy close to 94%.

**Figure 11:**
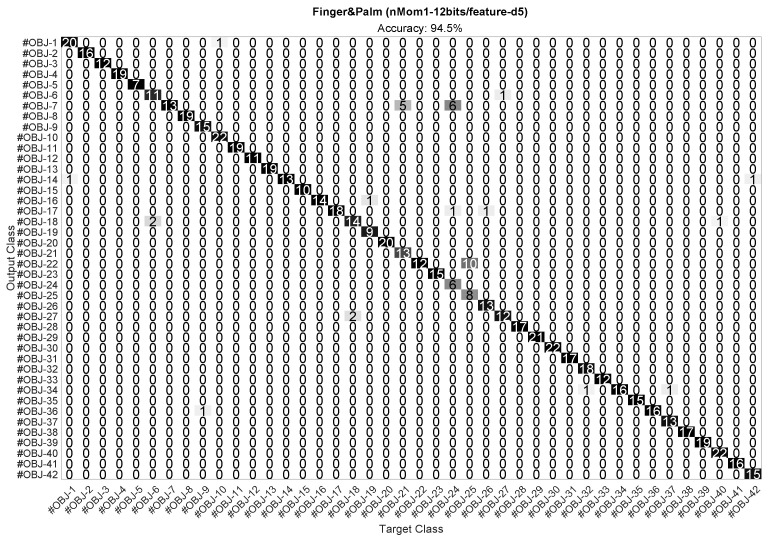
Confusion matrix of the optimal implementation in Figure 10. This case (second row of Table 4) used the first raw moment $M_{0,0}$ for both finger and palm, the d5 feature, and 12 bits per feature.

The confusion matrix for the best case Finger&Palm (nMom1-12bits/feature-d5) in Figure 10 is depicted in Figure 11. This confusion matrix shows that this implementation accurately classified the 42 objects in Figure 4, reaching an accuracy of 94.5% in our experiments. Despite the large size of the test set (673 feature vectors), there were only a few misclassifications.

With respect to the computation time, it was the same for all cases in Table 4 (three clock cycles after the last input vector was read, see Section 4), thanks to the parallel implementation illustrated in Figure 8.

### 5.2. Results Obtained with PCA

When PCA was applied, the accuracy results for the same cases of Table 3 were as shown in Table 5, where nCm means the number of principal components.

**Figure 12:**
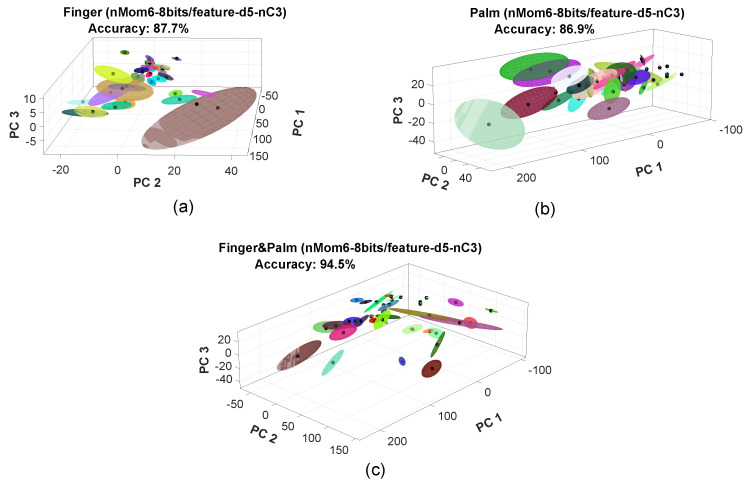
Comparison of a three principal component dimensional feature space for (**a**) finger, (**b**) palm, and (**c**) finger and palm. In all cases, the number of moments was six, the feature used was d5 from Table 2, and the number of bits per feature was 8. In the figure, PC1 stands for principal component 1, PC2 stands for principal component 2, and PC3 stands for principal component 3.

Figure 12 shows a comparison in three-dimensional feature space with three principal components, when the finger, palm, and finger–palm were used. In all cases, six moments were used, the d5 feature was applied, and there were 8 bits per feature. The training sets for each class in Figure 12 are represented as Gaussian ellipsoid distributions. The centroid of each class, a 3-value array, was determined through the training procedure outlined in Section 3.5. The classes in Figure 12c are more separated from each other than in Figure 12a,b; therefore, the classifier was more accurate when it received information from multiple sources. This can be seen in Table 5 and was also observed when PCA was not applied, as mentioned in the previous section.

As done when PCA was not applied, we selected the top-performing cases in terms of accuracy (bolded in Table 5). The results of the consumption of hardware resources in these cases are presented in Table 6. This time, the number of cycles the PCA computation took depended on the number of PCA components (see last column in Table 6). The consumption of memory resources was similar in all cases, which means that increasing the feature vector dimension did not imply a significant increment in hardware resources.

**Figure 13:**
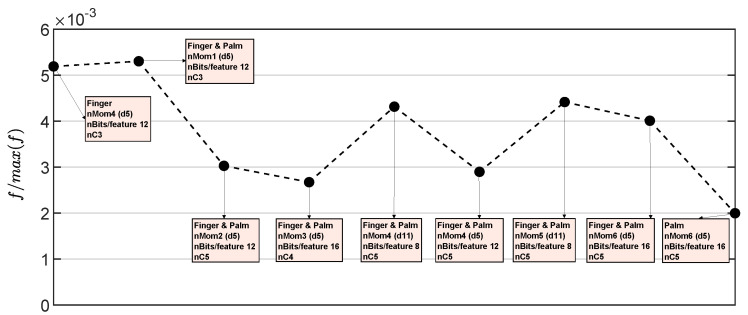
Result of the figure of merit in (2) normalized with respect to the best case in Figure 10.

**Figure 14:**
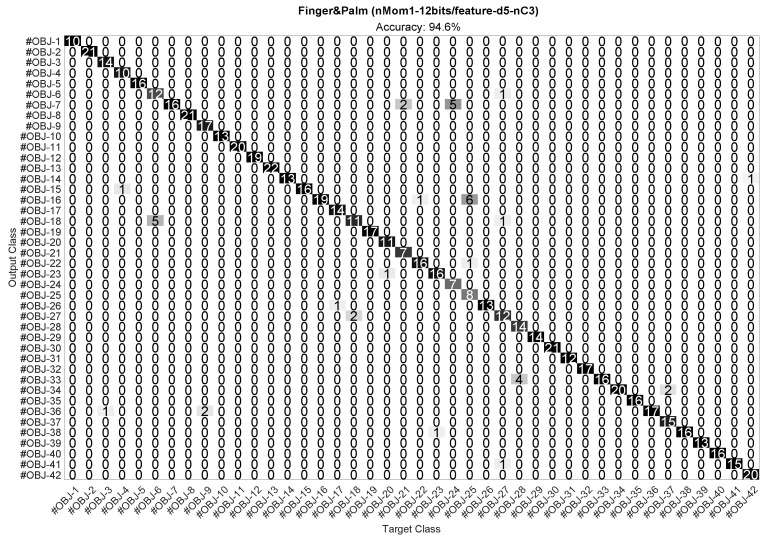
Confusion matrix of the optimal implementation in Figure 13. This case (second row of Table 6, used the first raw moment $M_{0,0}$ for both finger and palm, the d5 feature, and 12 bits per feature, and three principal components.

Figure 13 shows a comparison of the cases in Table 6 with the figure of merit defined in (2). The best case now was Finger&Palm, nMom1 (d5), nBits/feature 12, nC3 and its corresponding confusion matrix is displayed in Figure 14.

**Figure 15:**
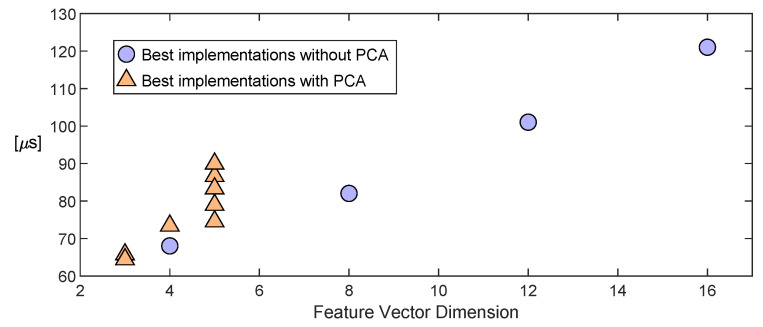
Feature extraction delay plus classification delay vs. the classifier input feature vector dimension. The results correspond to the cases in Table 4 and Table 6, for the implementations without PCA and with PCA, respectively.

Figure 15 depicts the result of adding the input–output delay of the feature extraction (see Table 4 and Table 6) to the delay of the classifier. The latter was measured with a MDO4104B-6 Mixed Domain Tektronix^®^ 6 GHz Oscilloscope. The dimension of the feature vector (number of features as components) is the variable on the x-axis of Figure 15. It can be seen that the larger the number of features, the longer it took for the classifier to provide an output. Since the number of features was lower in implementations with the PCA module, these realizations performed better for this aspect.

## 6. Conclusions

This paper proposes a strategy for recognizing the compliance of objects with tactile sensors. This strategy is intended to be implemented in smart sensors that have embedded electronics. Specifically, previously reported sensors with electronics based on FPGAs were mounted on the gripper. These sensors provide the raw moments of the tactile image, and they are registered when the target object is squeezed and desqueezed. Four parameters from the thus-obtained curve were proposed as input features for the classifier. Principal component analysis was also considered, to reduce the dimensions of the feature vector. Many different options were then analyzed, depending on the number of moments and features, and the size in bits of these features, in terms of the classification accuracy and performance of the realization in the Zynq™-7000 System on Chip. The feature extraction was carried out on the FPGA of the SoC, while the k-means classifier was implemented in its ARM core.

From the analysis of the results, it can be concluded that the best results in terms of classification accuracy were achieved when both finger and palm sensors were used (above 94% accuracy). In this case, the implementation of the feature extraction on the FPGA that ddid not apply PCA was much more efficient in terms of resources (see Table 4 and Table 6) and power consumption (2 mW versus 32 mW with PCA).

The application of PCA could be advantageous to reduce the input–output delay of the classifier and increase the separation between classes. This work was carried out with a large set of 42 classes, and the application of PCA provided a slight improvement in the input–output delay. Moreover, it is interesting to consider the case of using only the finger sensor, which simplified the system. In this case, if the first four raw moments of the tactile image were registered, the achieved classification accuracy was 91.7% without PCA and 90.9% with PCA and three components. Therefore, the classification accuracies were similar, although the consumption of resources by feature extraction was much higher when PCA was applied, and the power consumption of this part was 34 mW versus 4 mW without PCA. However, the time of the feature extraction plus the input–output delay of the classifier was 65.7 μs with PCA and 82 μs without PCA.

In summary, with the setup and set of classes used in this paper, the procedure that did not apply PCA was better, because the proposed strategy based on four simple features from the raw moments graphs resulted in a very efficient realization. An improvement in the input–output delay was observed with PCA, which was more significant if only the finger sensor was used. The choice between both options also depends on the complexity of other tasks that have to be carried out in real time.

Further works could be carried out considering different aspects. First, the influence of the object size and spatial resolution of the tactile sensors on the classification performance and consumption of resources should be assessed. Second, other learning and classification algorithms could be implemented and evaluated. Third, the capability for recognizing the object compliance has to be integrated with other functions, such as that of texture detection. Finally, the proposed procedure could be implemented in other commercial artificial hands and grippers equipped with tactile sensors.

## Figures and Tables

**Table 1:** Table with the forty-two classes employed in this work, also see Figure 4.

Object Label	Object Description	Exploring Position	Object Label	Object Description	Exploring Position
#OBJ-1	Avocado	Horizontal	#OBJ-22	Hand-therapy grip orange 3D ovoid	Face Down
#OBJ-2	Eggplant	Horizontal	#OBJ-23	Hand-therapy grip orange 3D ovoid	Face Up
#OBJ-3	Plum	Standard	#OBJ-24	Hand-therapy grip orange 3D ovoid	Horizontal
#OBJ-4	Foam cube	Vertical	#OBJ-25	Hand-therapy grip green 3D ovoid	Face Down
#OBJ-5	Foam cube	Vertical	#OBJ-26	Hand-therapy grip green 3D ovoid	Face Up
#OBJ-6	Hand-therapy grip sphere	Standard	#OBJ-27	Hand-therapy grip green 3D ovoid	Horizontal
#OBJ-7	Green Filaflex 3D printed sphere	Standard	#OBJ-28	Potato	Horizontal
#OBJ-8	Hydro-alcoholic gel	Horizontal	#OBJ-29	Paddle ball	Standard
#OBJ-9	Kiwi	Horizontal	#OBJ-30	Pear	Horizontal
#OBJ-10	Lettuce	Horizontal	#OBJ-31	TPU 3D printed pyramid	Face Down
#OBJ-11	Ripe lemon	Horizontal	#OBJ-32	TPU 3D printed pyramid	Face Up
#OBJ-12	Green lemon	Horizontal	#OBJ-33	Green banana	Horizontal
#OBJ-13	Ripe tangerine	Horizontal	#OBJ-34	Ripe banana	Horizontal
#OBJ-14	Green tangerine	Horizontal	#OBJ-35	Rotten banana	Horizontal
#OBJ-15	Rotten nectarine	Horizontal	#OBJ-36	Tomato	Horizontal
#OBJ-16	Hand-therapy grip blue 3D ovoid	Face Down	#OBJ-37	Filaflex 3D printed toroid	Horizontal
#OBJ-17	Hand-therapy grip blue 3D ovoid	Face Up	#OBJ-38	Filaflex 3D printed toroid	Vertical
#OBJ-18	Hand-therapy grip blue 3D ovoid	Horizontal	#OBJ-39	TPU 3D printed triangle	Face Down
#OBJ-19	Hand-therapy grip purple 3D ovoid	Face Down	#OBJ-40	TPU 3D printed triangle	Face Up
#OBJ-20	Hand-therapy grip purple 3D ovoid	Face Up	#OBJ-41	TPU 3D printed triangle	Horizontal
#OBJ-21	Hand-therapy grip purple 3D ovoid	Horizontal	#OBJ-42	Carrot	Horizontal

**Table 2:** Labels for the combination of features: area under the curve (AR), maximum value (MAX), magnitude value in the ascending part at 2/3 of the time needed to reach the maximum (H1) and magnitude value at 1/3 of the same time after the maximum was reached (H2).

Combination of Features	Label
AR	d1
MAX	d2
H1	d3
H2	d4
AR and MAX	d5
AR and H1	d6
AR and H2	d7
MAX and H1	d8
MAX and H2	d9
H1 and H2	d10
AR, MAX and H1	d11
AR, MAX and H2	d12
AR, H1 and H1	d13
MAX, H1 and H2	d14
AR, MAX, H1 and H2	d15

**Table 3:** Classification accuracy without PCA.

Combination of $M_{p,q}$ Moments							
Sensor	Nbits/Feature	$M_{0,0}$	$M_{0,0}$,$M_{0,1}$	$M_{0,0}$,$M_{0,1}$, $M_{1,0}$	$M_{0,0}$,$M_{0,1}$, $M_{1,0}$,$M_{0,2}$	$M_{0,0}$,$M_{0,1}$, $M_{1,0}$,$M_{0,2}$, $M_{2,0}$	$M_{0,0}$,$M_{0,1}$, $M_{1,0}$,$M_{0,2}$, $M_{2,0}$,$M_{1,1}$
	8	<23% (all cases)	<45% (all cases)	68.8% (d15)	82.3% (d15)	88.4% (d11)	89.1% (d5)
Finger	12	63.2% (d11)	80.5% (d5)	86.8% (d11)	91.7% **(d5)**	89.6% (d5)	90.5% (d5)
	16	75% (d11)	83.6% (d11)	88.5% (d11)	90.9% (d11)	90.2% (d5)	89.9% (d5)
	8	<50% (all cases)	79.2% (d11)	86.2% (d11)	86.9% (d11)	86.9% (d5)	89% (d5)
Palm	12	84.8% (d11)	87.5% (d5)	88.2% (d5)	88% (d5)	87.7% (d5)	89.3% (d5)
	16	82% (d5)	84.2% (d11)	88.4% (d5)	87.1% (d5)	87.8% (d5)	89.6% **(d5)**
	8	68.6% (d11)	93.3% (d11)	97.3% (d11)	95.5% **(d11)**	97.2% **(d11)**	98.5% **(d11)**
Finger and Palm	12	94.5% **(d5)**	96.4% **(d5)**	97% (d5)	95.5% **(d5)**	95.7% (d5)	98.2% (d5)
	16	93.5% (d5)	96.3% (d5)	97.5% **(d5)**	95.1% (d5)	96.1% (d5)	98.2% (d8)

**Table 4:** Performance data for the implementation of the feature extraction without PCA (VHDL features computing module in Figure 7), as provided by the real Vivado IDE^®^ Utilization Report. The last two columns are the input–output delay of the module (in ns), and its power consumption (in mW).

Sensor	Best Case	LUTRAM	BRAMs	FF Pairs	LUT Logic	DSPs	F-MUXES	$t_{\mathrm{feature}\mathrm{extraction}}$ (ns)	Power Consumption (mW)
Finger	$M_{0,0}$,$M_{0,1}$,$M_{1,0}$,$M_{0,2}$ d5, 12 bits/feature	0	0	14	19	0	0	30	4
Finger and Palm	$M_{0,0}$ d5, 12 bits/feature	0	0	28	38	0	0	30	2
Finger and Palm	$M_{0,0}$,$M_{0,1}$ d5, 12 bits/feature	0	0	56	76	0	0	30	4
Finger and Palm	$M_{0,0}$,$M_{0,1}$, $M_{1,0}$ d5, 16 bits/feature	0	0	108	136	0	0	30	6
Finger and Palm	$M_{0,0}$,$M_{0,1}$, $M_{1,0}$,$M_{0,2}$ d11, 8 bits/feature	0	0	72	128	0	0	30	12
Finger and Palm	$M_{0,0}$,$M_{0,1}$, $M_{1,0}$,$M_{0,2}$ d5, 12 bits/feature	0	0	112	152	0	0	30	8
Finger and Palm	$M_{0,0}$,$M_{0,1}$, $M_{1,0}$,$M_{0,2}$, $M_{2,0}$ d11, 8 bits/feature	0	0	90	160	0	0	30	15
Finger and Palm	$M_{0,0}$,$M_{0,1}$, $M_{1,0}$,$M_{0,2}$, $M_{2,0}$,$M_{1,1}$ d11, 8 bits/feature	0	0	108	192	0	0	30	18
Palm	$M_{0,0}$,$M_{0,1}$, $M_{1,0}$,$M_{0,2}$, $M_{2,0}$,$M_{1,1}$ d5, 16 bits/feature	0	0	84	114	0	0	30	6

**Table 5:** Classification accuracy with PCA.

Combination of $M_{p,q}$ Moments							
Sensor	Nbits/Feature	$M_{0,0}$	$M_{0,0}$,$M_{0,1}$	$M_{0,0}$,$M_{0,1}$, $M_{1,0}$	$M_{0,0}$,$M_{0,1}$, $M_{1,0}$,$M_{0,2}$	$M_{0,0}$,$M_{0,1}$, $M_{1,0}$,$M_{0,2}$, $M_{2,0}$	$M_{0,0}$,$M_{0,1}$, $M_{1,0}$,$M_{0,2}$, $M_{2,0}$,$M_{1,1}$
	8	<30% (all cases)	<45% (all cases)	64.4% (d15, nC5)	81.4% (d11, nC5)	85.9% (d11, nC5)	89% (d11, nC6)
Finger	12	64.1% (d15, nC4)	83% (d5, nC3)	89.3% (d5, nC4)	90.9% **(d5, nC3)**	90.6% (d5, nC6)	91.1% (d5, nC5)
	16	76.2% (d5, nC2)	85.3% (d5, nC4)	90.5% (d5, nC4)	91.2% (d5, nC6)	90.5% (d11, nC6)	90.8% (d5, nC4)
	8	<41% (all cases)	75.2% (d11, nC4)	85.2% (d5, nC6)	87.2% (d5, nC6)	88% (d5, nC6)	89.4% (d5, nC5)
Palm	12	84.5% (d11, nC3)	86.5% (d5, nC4)	88.5% (d5, nC6)	89% **(d5, nC5)**	87.1% (d5, nC5)	89.1% (d5, nC6)
	16	82.7% (d11, nC3)	87.5% (d5, nC3)	88.2% (d11, nC6)	88.5% (d5, nC5)	87.8% (d5, nC5)	89% (d5, nC5)
	8	64.7% (d15, nC4)	92% (d11, nC5)	95.8% (d11, nC4)	94.5% **(d11, nC5)**	96.3% **(d11, nC5)**	94.4% **(d11, nC3)**
Finger and Palm	12	94.6% **(d5, nC3)**	97.3% **(d5, nC5)**	97.3% (d5, nC5)	95.2% **(d5, nC5)**	96.1% (d5, nC5)	95.5% (d8, nC3)
	16	95.1% (d5, nC4)	96.9% (d5, nC5)	97% **(d11, nC5)**	95.1% (d5, nC6)	96.9% (d5, nC6)	97% (d8, nC5)

**Table 6:** Performance data for the implementation of the feature extraction with PCA (VHDL features computing module plus HLS principal component analysis (PCA) computing module in Figure 7), as provided by the real Vivado IDE^®^ Utilization Report. The columns of the Table are the same as in Table 4.

Sensor	Best PCA Case	LUTRAM	BRAMs	FF Pairs	LUT Logic	DSPs	F-MUXES	$t_{\mathrm{feature}\mathrm{extraction}}$ (μs)	Power Consumption (mW)
Finger	$M_{0,0}$,$M_{0,1}$, $M_{1,0}$,$M_{0,2}$ d5, 12 bits/feature, nC3	40	2	441	1076	7	6	2.75	34
Finger and Palm	$M_{0,0}$ d5, 12 bits/feature, nC3	36	2	443	1078	7	6	1.43	32
Finger and Palm	$M_{0,0}$,$M_{0,1}$ d5, 12 bits/feature, nC5	36	3	454	1110	7	6	4.55	37
Finger and Palm	$M_{0,0}$,$M_{0,1}$, $M_{1,0}$ d5, 16 bits/feature, nC4	48	3	516	1219	7	6	5.41	39
Finger and Palm	$M_{0,0}$,$M_{0,1}$, $M_{1,0}$,$M_{0,2}$ d11, 8 bits/feature, nC5	36	3	478	1185	7	6	13.35	46
Finger and Palm	$M_{0,0}$,$M_{0,1}$, $M_{1,0}$,$M_{0,2}$ d5, 12 bits/feature, nC5	36	3	518	1198	7	6	8.95	41
Finger and Palm	$M_{0,0}$,$M_{0,1}$, $M_{1,0}$,$M_{0,2}$, $M_{2,0}$ d11, 8 bits/feature, nC5	36	3	507	1235	7	6	16.65	49
Finger and Palm	$M_{0,0}$,$M_{0,1}$, $M_{1,0}$,$M_{0,2}$, $M_{2,0}$,$M_{1,1}$ d11, 8 bits/feature, nC5	24	4	495	1310	7	6	19.95	54
Palm	$M_{0,0}$,$M_{0,1}$, $M_{1,0}$,$M_{0,2}$, $M_{2,0}$,$M_{1,1}$ d5, 16 bits/feature, nC5	48	3	506	1202	7	6	13.35	40

## Data Availability

The processing codes and data segments can be obtained by contacting the corresponding author.
